# Stability and Degradation of Caffeoylquinic Acids under Different Storage Conditions Studied by High-Performance Liquid Chromatography with Photo Diode Array Detection and High-Performance Liquid Chromatography with Electrospray Ionization Collision-Induced Dissociation Tandem Mass Spectrometry

**DOI:** 10.3390/molecules21070948

**Published:** 2016-07-21

**Authors:** Meng Xue, Hang Shi, Jiao Zhang, Qing-Quan Liu, Jun Guan, Jia-Yu Zhang, Qun Ma

**Affiliations:** 1 School of Chinese Pharmacy, Beijing University of Chinese Medicine, Beijing 100102, China; xuemeng0421@163.com (M.X.); shhang2012@sina.cn (H.S.); zhangjiao1st@163.com (J.Z.); timeguan@sina.com (J.G.); 2 Beijing Hospital of Traditional Chinese Medicine, Beijing 100010, China; liuqingquan2003@126.com; 3 Beijing Institution of Chinese Medicine, Beijing University of Chinese Medicine, Beijing 100029, China

**Keywords:** caffeoylquinic acids (CQAs), stability, temperature, light irradiation, solvent, HPLC-MS^n^

## Abstract

Caffeoylquinic acids (CQAs) are main constituents in many herbal medicines with various biological and pharmacological effects. However, CQAs will degrade or isomerize when affected by temperature, pH, light, etc. In this study, high-performance liquid chromatography with photodiode array detection (HPLC-PDA) and high-performance liquid chromatography tandem mass spectrometry (HPLC-MS/MS) was utilized to study the stability and degradation of CQAs (three mono-acyl CQAs and four di-acyl CQAs) under various ordinary storage conditions (involving different temperatures, solvents, and light irradiation). The results indicated that the stability of CQAs was mainly affected by temperature and light irradiation, while solvents did not affect it in any obvious way under the conditions studied. Mono-acyl CQAs were generally much more stable than di-acyl CQAs under the same conditions. Meanwhile, the chemical structures of 30 degradation products were also characterized by HPLC-MS^n^, inferring that isomerization, methylation, and hydrolysis were three major degradation pathways. The result provides a meaningful clue for the storage conditions of CQAs standard substances and samples.

## 1. Introduction

Chlorogenic acids (CGAs) are a family of natural phenolic compounds, named by Clifford in 1985, that including caffeoylquinic acids (CQAs), *p*-coumaroylquinic acids (*p*-CoQAs) and feruloyl quinic acids (FQAs), etc. [1]. They are formed by esterification of quinic acid with caffeic acids [2,3,4]. They are the most common CGAs and can be found in a wide variety of consumer goods, including vegetables, fruits, and herbs [5]. CQAs have various kinds of biological and pharmacological effects, such as antioxidant, anti-carcinogenic, and antihypertensive activities [6,7]. Previous reports revealed that 5-CQA was unstable at high temperatures [8,9,10]. For example, the heating of 5-CQA in the temperature range of 100–200 °C caused isomerization and other transformations [11]. Meanwhile, 3-CQA and 3,5-dicaffeoylquinic acids (3,5-diCQA) are stable under acidic conditions, while at neutral and basic pH values, isomerization of 3-CQA to 4-CQA/5-CQA, as well as the isomerization of 3,5-diCQA to 3,4-diCQA and 4,5-diCQA occurred rapidly [12]. Apparently, extreme conditions such as high temperature or acidic conditions have a great influence on the stability of some CQAs. However, the study of stability of mono- and di-acyl CQAs will be much more realistically meaningful and significant because these standard substances are widely applied in quality control of traditional Chinese medicines under ordinary storage conditions.

**Figure 1:**
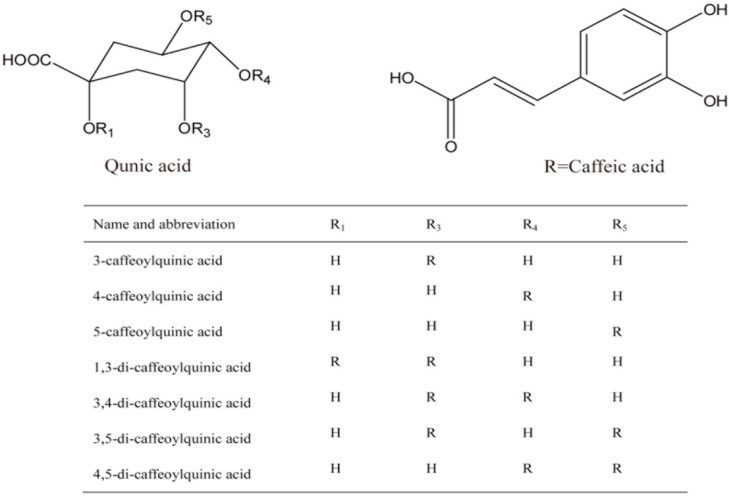
The structures of the seven mono- and di-acyl CQA standards.

In this paper, a stability study of CQAs (including three mono-acyl CQAs and four di-acyl CQAs, shown in Figure 1) under simulated storage conditions was performed using high-performance liquid chromatography with PDA detection (HPLC-PDA) and liquid chromatography tandem mass spectrometry (LC-MS/MS). Our study examined the thermal-, photo-, and solvent-stability of mono- and di-acyl CQAs as well as analysis of their degradation products. Degradation products of seven different CQAs (stored at room temperature and in transparent bottles) were also characterized by employing the established LC-MS method.

## 2. Results and Discussion

### 2.1. Method Validation

**Figure 2:**
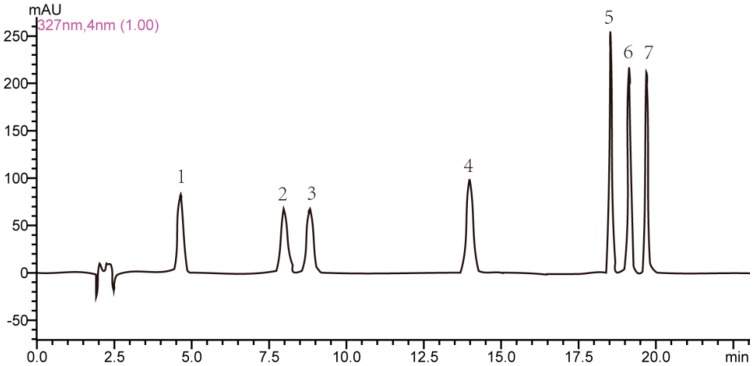
HPLC-PDA chromatogram of (1) 5-caffeoylquinic acid; (2) 3-caffeoylquinic acid; (3) 4-caffeoylquinic acid; (4) 1,3-dicaffeoylquinic acid; (5) 3,4-dicaffeoylquinic acid; (6) 3,5-dicaffeoylquinic acid; (7) 4,5-dicaffeoylquinic acid.

A representative HPLC-PDA chromatogram of a mixed standard solution of seven CQAs separated under the optimized chromatography and detection conditions is shown in Figure 2. The method validation assays were carried out under the optimized conditions including the linearity, limits of detection (LOD), lower limit of quantification (LLOQ), precision, repeatability, and recovery. The calibration curve for each compound was obtained with at least five appropriate concentrations. The regression equations for the seven CQAs were calculated in the form of *y = ax + b*, where *y* and *x* were the peak area and the corresponding sample quantity of CQAs injected, respectively. As shown in Table 1, the correlation coefficients of all target components exceeded 0.9993 with good linearity. The limits of detection (LODs) and lower limits of quantitation (LLOQs) under the present chromatographic conditions were determined at a signal-to-noise ratio (S/N) of 3 and 10 by series dilution from stock solution with LODs ranged from 0.10 µg/mL to 0.40 µg/mL and LLOQs ranged from 0.34 µg/mL to 1.32 µg/mL, respectively.

For the precision test, the mixed standard solutions were analyzed for six replicates within a day. Good precision was shown in Table 2, for Relative Standard Deviation (RSD) of peak areas for each compound was not more than 0.56%. The RSD% was also taken as measures of repeatability. The result of repeatability study was reported in Table 2, shown that RSD% values of seven compounds were less than 1.40%. The results obtained for precision and repeatability were summarized in Table 2.

### 2.2. Thermal-Stability, Photo-Stability, and Solvent-Stability Study

Thermal-stability, photo-stability, and solvent-stability were studied to investigate effects of ordinary storage conditions. The thermal-stability of CQAs was studied at 4 °C and room temperature (25 °C) in seven consecutive days. Samples were kept in transparent glass and brown glass respectively to study photo-stability of CQAs. The influence of solvent was studied as well.

#### 2.2.1. Thermal-Stability Study

**Figure 3:**
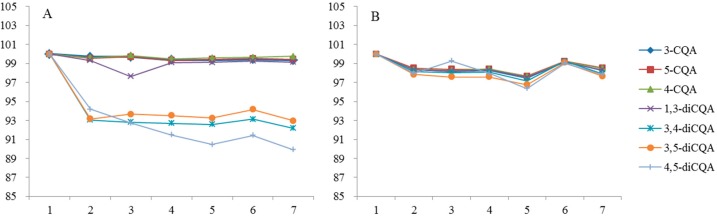
Thermal stability of CQAs in solution of 50% (*v*/*v*) aqueous methanol stored at brown bottle. (**A**) Room temperature; (**B**) 4 °C.

In the thermal stability study (Figure 3), CQAs were relatively stable at 4 °C for 7 days, but degraded extensively at room temperature. Mono-acyl CQAs were stable at room temperature while di-acyl CQAs except 1,3-diCQA were all decreased. This can possibly be attributed to the fact that meta-substituted 1,3-diCQA without a 2-hydroxy group offer less steric hindrance. However, it is not fully clear at this stage and more studies are needed.

**Scheme 1:**
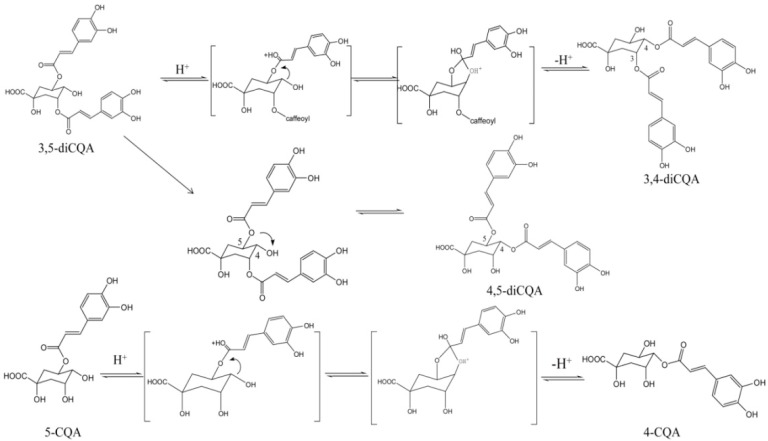
Acyl migration mechanism of 4,5-diCQA ⇋ 3,5-diCQA ⇋ 3,4-diCQA and 5-CQA ⇋ 4-CQA ⇋ 3-CQA.

About 10.08% of 4,5-diCQA, 7.82% of 3,4-diCQA, and 7.03% of 3,5-diCQA degraded after 7 days, which suggested that temperature has a great effect on the stability of di-acyl CQAs. 4,5-diCQA was much more stable than 3,4-diCQA and 3,5-diCQA, which is similar with the results reported by Y Li et al.: mono-acyl CQAs are much more stable than di-CQAs under heating condition, because the mechanism of 5-CQA ⇋ 4-CQA ⇋ 3-CQA is acyl migration and the mechanism of 4,5-diCQA ⇋ 3,5-diCQA ⇋ 3,4-diCQA is similar to mono-acyl CQAs [13,14]. The acyl migration mechanism of mono- and di-acyl CQAs is shown in Scheme 1. This might be due to the fact that di-acyl CQAs are more stable when the ester bond link to the quinic acid exists as an equatorial bond rather than an axial one [15]. Only one ester bond of 3,4-diCQA and 3,5-diCQA exist as an equatorial bond while all ester bonds of 4,5-diCQA exist as equatorial bonds.

#### 2.2.2. Photo-Stability Study

**Figure 4:**
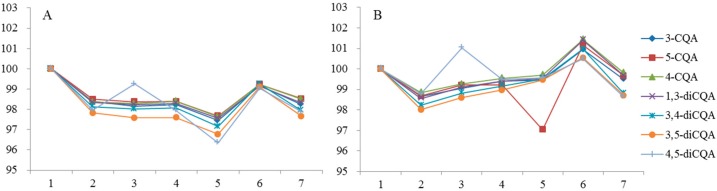
Photostability of CQAs in solution of 50% (*v*/*v*) aqueous methanol stored at 4 °C. (**A**) Brown bottle; (**B**) Transparent bottle.

The remaining percentage of CQAs in the photostability studies (Figure 4) showed that the relative contents of CQAs fluctuated between 95% and 105% during seven days. The fluctuation was probably because light accelerated the acyl migration (Scheme 1), which led to mutual conversion of CQAs.

#### 2.2.3. Solvent-Stability Study

**Figure 5:**
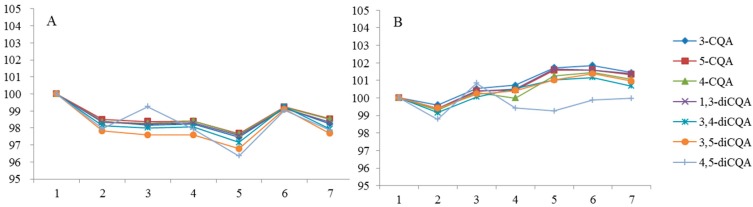
Stability of CQAs of different solvent in brown bottle at refrigerator. (**A**) 50% (*v*/*v*) aqueous methanol; (**B**) Methanol.

**Figure 6:**
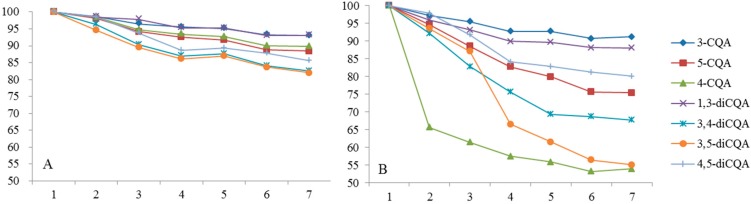
Stability of CQAs in transparent bottle at room temperature. (**A**) 50% (*v*/*v*) aqueous methanol; (**B**) Methanol.

The study of the stability of CQAs in two solvents (methanol and 50% (*v*/*v*) aqueous methanol) kept in brown glass bottles at 4 °C (Figure 5) showed slow degradation of the compounds in two solvents. Meanwhile, noticeable decreases in concentrations of CQAs were observed when kept in transparent glass at room temperature (Figure 6). As presented in Figure 6A, about 18.02% of 4,5-diCQA, 17.44% of 3,4-diCQA, 14.43% of 3,5-diCQA, 6.89% of 1,3-diCQA, 6.96% of 4-CQA, 10.19% of 5-CQA, and 11.59% of 3-CQA were degraded in 50% methanol solution. About 44.96% of 4,5-diCQA, 33.25% of 3,4-diCQA, 17.44% of 3,5-diCQA, 11.93% of 1,3-diCQA, 46.09% of 4-CQA, 24.63% of 5-CQA, and 8.82% of 3-CQA were degraded in 100% methanol solution (Figure 6B). CQAs degraded easily in methanol probably because increasing methanol in methanol/water solution causes an increase in the concentrations of the respective adducts or esters of methanol. 

The stabilities of CQAs differed under different conditions. Single factors such as temperature, light and solvent have little effect on the stabilities of mono-acyl CQAs. The thermal stability study of CQAs showed that apart from 1,3-diCQA, di-acyl CQAs showed poor stability under room temperature storage conditions. Steric hindrance of ester groups might be the main reason that mono-acyl CQAs were much more stable than di-CQAs. Di-acyl CQAs were more stable when the ester bond was linked to the meta-quinic acid. In addition, equatorial ester bonds will increase the stability of molecules. Light irradiation will not cause a decrease of CQAs but could lead to fluctuations of relative content of CQAs. The solvent stability study showed that CQAs were unstable when dissolved in methanol. Under dual factors of light and temperature, both mono-acyl CQAs and di-acyl CQAs decompose easily. Significant degradation was observed from samples stored at room temperature in methanol exposed to the light after 7 days, suggesting that CQAs should be stored in lower temperature (refrigerator) and light irradiation avoided.

### 2.3. Degradation Products Analysis

**Figure 7:**
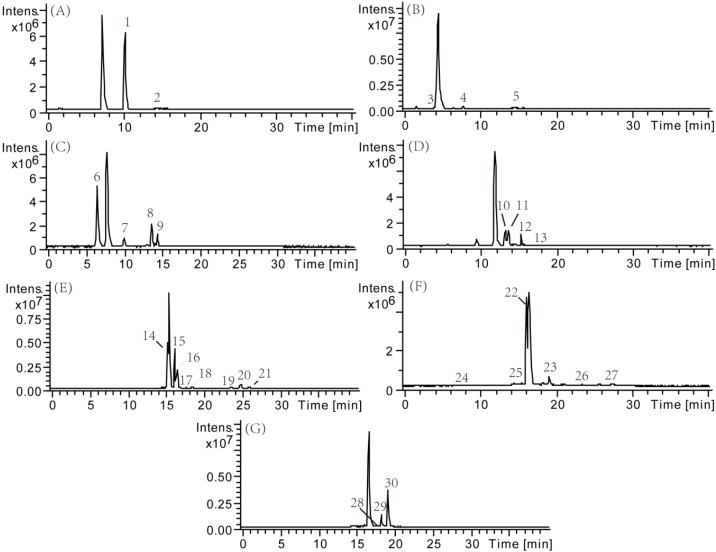
Total ion current chromatograms of 7 CQAs and its degradation products stored in transparent bottle at room temperature after 7 days. (**A**) 5-caffeoylquinic acid; (**B**) 3-caffeoylquinic acid; (**C**) 4-caffeoylquinic acid; (**D**) 1,3-dicaffeoylquinic acid; (**E**) 3,4-dicaffeoylquinic acid; (**F**) 3,5-dicaffeoylquinic acid; (**G**) 4,5-dicaffeoylquinic acid.

CQAs have poor stabilities and therefore are prone to generating many products in the experiments. The thermal and photo degradation products of samples (at room temperature and under light irradiation) were analyzed by the LC-MS^n^ method and confirmed by the comparison of LC behavior and MS^n^ data with reference standards and references. The major constituents were well detected and most of the investigated compounds exhibited [M − H]^−^ ions and product ions with rich structural information in the collision-induced dissociation tandem mass spectrometry experiment. The total ion current chromatograms (TICC) are presented in Figure 7 and the electrospray ionization tandem mass (ESI-MS/MS) data and fragment ions and relative content (obtained by comparing the peak area of each compound on the seventh day to the corresponding reference standard on the first day) of these related compounds were listed in Table 3. 

As a result, a total of eight degradation products of 3,4-diCQA, six degradation products of 3,5-diCQA, four degradation products of 1,3-diCQA, and three degradation products of 4,5-diCQA were identified in di-acyl CQA samples. Compared with di-acyl CQAs, mono-acyl CQAs were more stable, which coincided with our previous studies. Only two degradation products of 3-CQA, three degradation products of 5-CQA, and four degradation products of 4-CQA were detected and identified in mono-acyl CQA samples. 

#### 2.3.1. Degradation Products of Mono-Acyl CQAs

**Figure 8:**
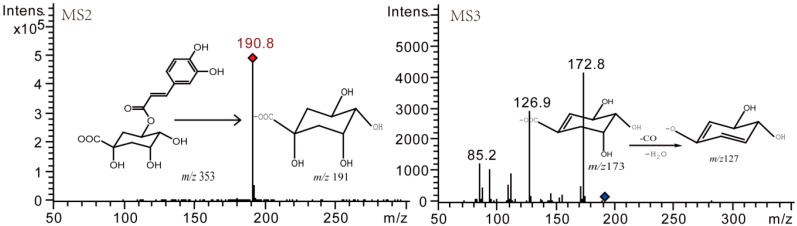
Negative MS^n^ spectra and fragment ions of compound **1**.

The primary degradation pathways of mono-acyl CQAs were isomerization, methylation, and dehydration. The fragment ions such as *m*/*z* 353 corresponding to [M − H]^−^, *m*/*z* 191 (C_7_H_11_O_6_^−^) corresponding to [quinic acid − H]^−^, and *m*/*z* 173 (C_7_H_9_O_5_^−^) corresponding to [quinic acid − H − H_2_O]^−^ could be regarded as the diagnostic ions of mono-acyl CQAs and their isomerization products [16]. For instance, compound **1**, a degradation product of 5-CQA, gave the prominent [M − H]^−^ ion at *m*/*z* 353 in its ESI-MS spectrum. It produced MS^2^ base peak ion at *m*/*z* 191 and MS^3^ base peak ion at *m*/*z* 173. Both isomers have the similar MS^n^ fragment ions and ion intensities. According to the previous report, *cis*-isomer were reported to be much more hydrophobic and elute later than their corresponding *trans*-isomers [17]. Therefore, compound **1** was tentatively assigned as *cis*-5-CQA (Figure 8). Similarly, compound **3** (a degradation product of 3-CQA) and compound **6** (a degradation product of 4-CQA) were identified as *cis*-3-CQA and *cis*-4-CQA, respectively. Meanwhile, the relative content of isomerization productions was only next to that of prototype (Table 2), indicating that substitution isomerism is a common phenomenon for mono-acyl CQAs.

**Figure 9:**
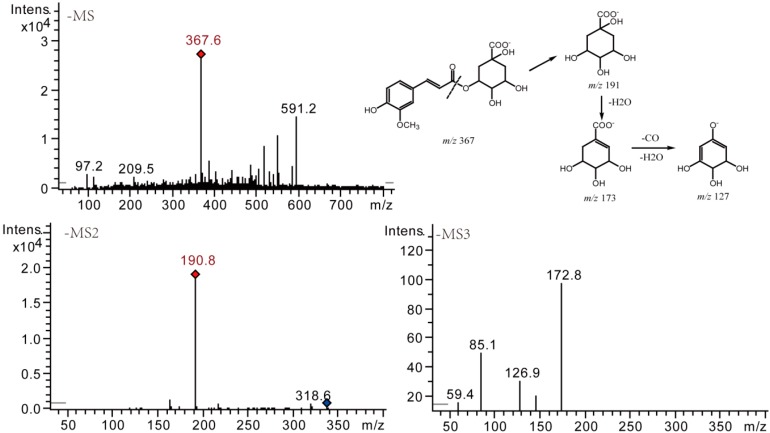
Negative MS^n^ spectra and proposed fragmentation patterns of compound **2**.

Methylation was another major degradation pathway when mono-acyl CQAs were stored in methanol. Methylated mono-acyl CQAs were detected from 5-CQA and 4-CQA solutions after 7 days. Here we took compound **2** (a degradation product of 5-CQA) as an example to describe the details (shown in Figure 9). The difference between the ions at *m*/*z* 367 and *m*/*z* 353 was 14 Da, indicating an adding of CH_2_ group to their prototypes which afforded [M − H]^−^ ion at *m*/*z* 353. Based on their retention times and ESI-MS/MS fragmentation, compounds **2**, **7**, and **8** were assigned as methylated 5-CQA and methylated 4-CQA, respectively.

**Figure 10:**
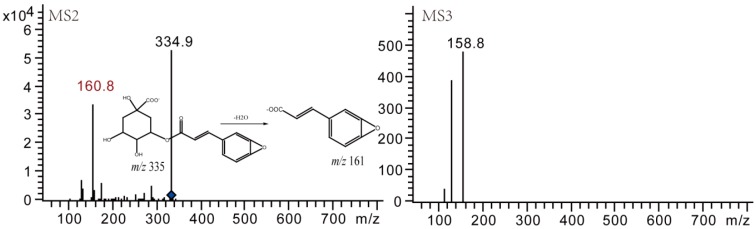
Negative MS^n^ spectra and fragment ions of compound dehydrated products.

Dehydrated production of mono-acyl CQAs were detected by LC-MS as well. Both degradation products of 3-CQA and 4-CQA afforded their prominent [M − H]^−^ ion at *m*/*z* 335 and MS^2^ base peak ion at *m*/*z* 161. The difference between the [M − H]^−^ ions at *m*/*z* 335 and quasi-molecular ion of their prototypes at *m*/*z* 353 was 18 Da, indicating the neutral loss of a H_2_O (Figure 10). Similarly, compounds **5** and **9** were finally identified as dehydrated 3-CQA and dehydrated 4-CQA, respectively [18]. 

#### 2.3.2. Degradation Products of Di-Acyl CQAs

**Figure 11:**
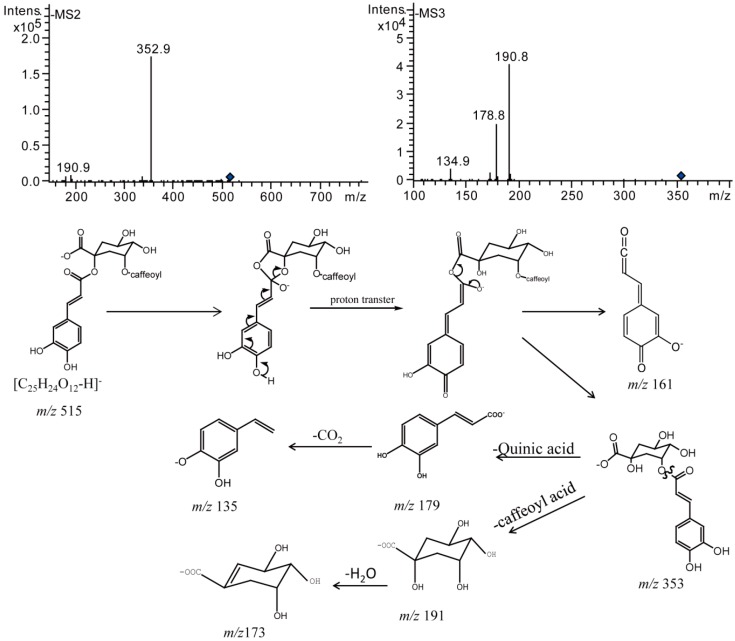
Negative MS^n^ spectra and proposed fragmentation patterns of compound **10**.

Di-acyl CQAs could produce more varieties of *cis*-isomers during the ordinary storage process. This might be due to the fact that di-acyl CQAs have one more caffeoyl compared with mono-acyl CQAs. All the di-acyl CQAs except 3,5-diCQA generated three categories of *cis*-isomers. Here we take 1,3-diCQA as an example to elaborate on the mass fragmentation patterns of isomerization products (Figure 11). 1,3-diCQA afforded [M − H]^−^ ion at *m*/*z* 515 (C_25_H_23_O_12_^−^), which was subsequently dissociated to generate MS^2^ base peak ion at *m*/*z* 353 (C_16_H_17_O_9_^−^) corresponding to [M − H − caffeoyl]^−^. It produced a MS^3^ base peak ion at *m*/*z* 191 (C_7_H_11_O_6_^−^) corresponding to [M − H – 2 caffeoyl]^−^ and significant fragment ion at *m*/*z* 135 corresponding to [caffeic acid − H − CO_2_]^−^. Compounds **10**–**12** have similar MS^n^ fragment ions and ion intensities compared with 1,3-diCQA, all of which were eluted later than 1,3-diCQA. Therefore, compound **12** was tentatively characterized as a di-*cis* isomer, while compounds **10** and **11** were identified to be mono-*cis* isomers according to their retention times and previous studies [17,19]. In the same way, *cis*-isomers of the other di-acyl CQAs were also identified (Table 2).

**Figure 12:**
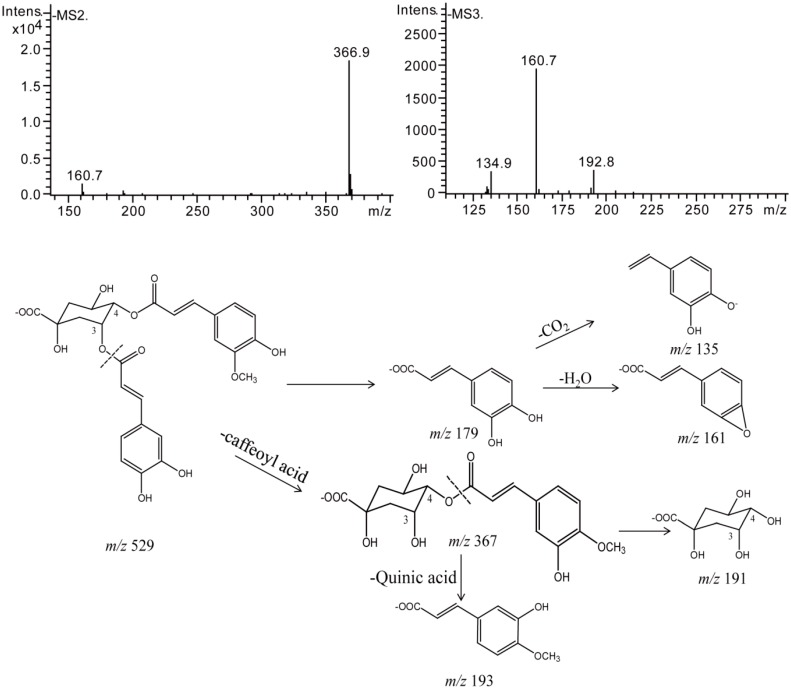
Negative MS^n^ spectra and proposed fragmentation patterns of Methlated 3,4-diCQA.

Besides isomerization products, both samples of 3,4-diCQA and 3,5-diCQA produced methylation products after 7 days when stored in transparent bottles at room temperature. Compounds **17** and **18** gave their respective prominent [M − H]^−^ ions at *m*/*z* 529, indicating an addition of a CH_2_ group to their prototypes which afforded prominent [M − H]^−^ ions at *m*/*z* 515. The difference between their MS^2^ base peak ion at *m*/*z* 367 and *m*/*z* 515 was 162 Da, which indicated the loss of a caffeoyl unit. They produced the MS^3^ peak ions at *m*/*z* 161, *m*/*z* 193, and *m*/*z* 135, which were characteristic of the methyl compounds and were assigned as methylated 3,4-diCQA (Figure 12). Similarly, compound **26** was assigned as methylated-3,5-diCQA. Meanwhile, compound **27** was identified as ethylated-3,5-diCQA, which gave a prominent [M − H]^−^ ion at *m*/*z* 543 (added a C_2_H_4_ group compared with its prototype) and product ions at *m*/*z* 381, *m*/*z* 161, *m*/*z* 179, and *m*/*z* 135 in its MS^2^ and MS^3^ spectra.

**Figure 13:**
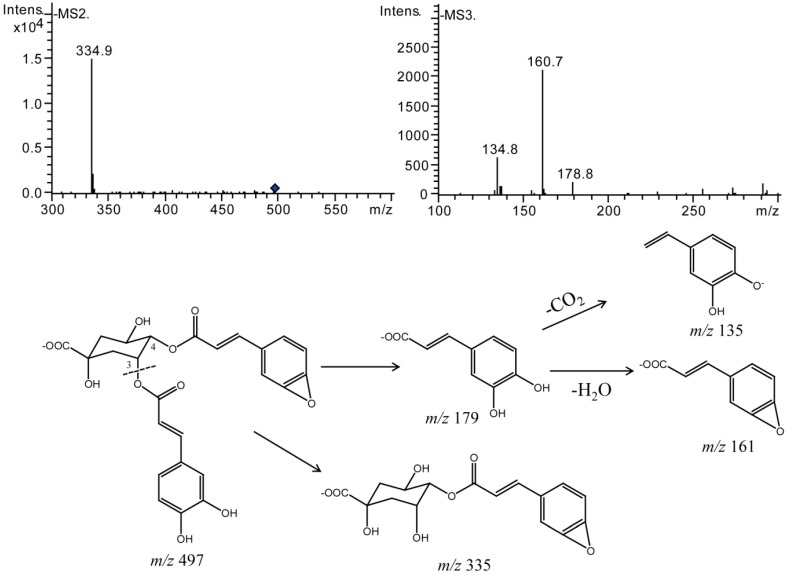
Negative MS^n^ spectra and proposed fragmentation patterns of dehydrated 3,4-diCQA.

Theoretically, there are dehydration products available in di-acyl CQAs solutions. However, only 3,4-diCQA produced dehydration products under the dual factor of light and temperature in our study. Compounds **19–21** displayed prominent [M − H]^−^ ions at *m*/*z* 497, indicating the losses of a H_2_O from their prototypes which afforded [M − H]^−^ ion at *m*/*z* 515, all of which produced the MS^2^ base peak ion at *m*/*z* 335 and MS^3^ product ions at *m*/*z* 161, *m*/*z* 197, and *m*/*z* 135. Therefore, they were assigned as dehydrated 3,4-diCQA (Figure 13).

## 3. Experimental Section

### 3.1. Chemicals and Materials

Acetonitrile, methanol, and formic acid are of HPLC grade (Fisher Scientific, Fairlawn, NJ, USA). The water used was purified by a Milli-Q system (Millipore, Milford, MA, USA). Seven standards including 5-CQA, 3-CQA, 4-CQA, 1,3-diCQA, 3,4-diCQA, 3,5-diCQA, and 4,5-diCQA were all purchased from National Institutes for Food and Drug Control (Beijing, China), and identified in our laboratory for qualitative analysis. All data for CQAs presented used the recommended IUPAC numbering system [17].

### 3.2. Preparation of Samples and Mixed Standard Solutions

To study different storage conditions, seven single-standard samples were obtained including 5-CQA, 4-CQA, 3-CQA, 1,3-diCQA, 3,5-diCQA, 3,4-diCQA, and 4,5-diCQA with methanol or 50% (*v*/*v*) aqueous methanol as solutions for concentrations of 39.37, 44.37, 38.57, 45.02, 35.09, and 44.06 μg/mL, respectively. The mixed standard solution of the above compounds was prepared in methanol to optimize the chromatography conditions. All samples were filtered through 0.22 μm membrane and 10 μL was directly injected into the HPLC system. 

### 3.3. HPLC Conditions

The analysis was performed on a Shimadzu HPLC system (Shimadzu Corporation, Kyoto, Japan) consisting of a quaternary pump, an autosampler, a photodiode array detector, and a column temperature controller. The data analysis was performed using the Shimadzu “LC Lab-Solution” software (Shimadzu Corporation). The samples were separated on an Agilent Zorbax Eclipse SB-C18 column (150 × 4.6 mm, 5 μm). The mobile phase consisted of acetonitrile (A) and water containing 0.1% formic acid (B), which was run with the gradient as follows: 0 min, 90% B; 10 min, 85% B; 11 min, 75% B; 25 min, 75% B; 26 min, 90% B; 40 min, 90% B. The flow rate was 0.8 mL/min and peaks were detected at 327 nm. The column temperature was set at 30 °C. The same elution conditions were used for the LC/MS experiments.

### 3.4. HPLC-DAD-ESI-MS/MS Analysis

For ESI-MS/MS analysis, an MSD Trap XCT Plus Mass spectrometer (Agilent Technologies, Santa Clara, CA, USA) was connected to the Agilent 1100 Series liquid chromatograph system, equipped with a binary pump, an auto sampler, a photo-diode array detector and a column temperature controller via an electrospray ionization (ESI) interface. Agilent 6300 Series Ion Trap LC/MS System 6.1 SR1 software (Angilent Technologies) was used for data control and management. Samples were analyzed in the negative ion mode with a tune method set as follows: nebulizer gas pressure of 40.00 psi; dry gas flow rate of 11.00 L/min; electrospray voltage of the ion source of 3500 V; capillary temperature of 350 °C; capillary exit of 121.0 V; skimmer of 40.0 V; compound stability of 50%; trap drive level of 100%; target mass of *m*/*z* 400; scan range of *m*/*z* 100–800; AutoMS (4) operation mode; collision energy of 1 V; SmartFrag start ampl of 30%, SmartFrag end ampl of 200%. As required, more sensitive targeted MS^n^ experiments were also used to seek compounds with a particular molecular ion that might otherwise have been overlooked, e.g., *m*/*z* 353 to seek mono-acyl caffeoylquinic acids (CQAs), *m*/*z* 515 to seek di-acyl caffeoylquinic acids (DiCQAs).

## 4. Conclusions

In the present study, we report how three major factors (temperature, solvent, and light irradiation) affect the stability of CQAs according to practical circumstances. A sensitive and rapid LC-MS assay was established for the analysis of degradation products of CQAs. From this experiment we have identified 30 different kinds of degradation products, and therefore provided useful clues for production and quality control. The results showed that all CQAs decompose easily under the dual factor influence of light and temperature. The chemical structures of degradation products were characterized by LC-MS, and suggested that isomerization, methylation, and hydrolysis were three possible degradation pathways. Our study will provide meaningful data for the ordinary storage conditions of CQA standard substances and samples. 

## Figures and Tables

**Table 1:** Regression data, limits of detection (LODs), lower limit of quantification (LLOQs) for seven CQAs.

Compounds	Liner and Range			LOD and LLOQ	
	Range (μg)	*y = ax + b*	r^2^	LOD ^a^ (µg/mL)	LLOQ ^b^ (µg/mL)
5-CQA	0.102–1.102	*y =* 107454*x +* 34148	0.9993	0.20	0.66
4-CQA	0.067–1.340	*y =* 157825*x +* 17890	0.9999	0.25	0.83
3-CQA	0.044–0.888	*y =* 81939*x +* 35839	0.9999	0.40	1.32
1,3-diCQA	0.254–1.278	*y =* 117069*x −* 1843.9	0.9998	0.25	0.82
3,5-diCQA	0.213–1.068	*y =* 200743*x +* 13975	0.9996	0.12	0.39
3,4-diCQA	0.117–1.175	*y =* 284949*x +* 6894	0.9998	0.10	0.34
4,5-diCQA	0.095–0.950	*y =* 138795*x −* 2935.6	0.9998	0.17	0.55

^a^ LOD (µg/mL) = 3 × C (µg/mL)/(S/N); ^b^ LLOQ (µg/mL) = 3.3 × LOD.

**Table 2:** Precision and repeatability for the assay of seven CQAs.

Compounds	Precision (*n* = 6)		Repeatability (*n* = 6)	
	Peak Area	RSD (%)	Peak Area	RSD (%)
	(Mean ± SD)		(Mean ± SD)	
5-CQA	1,136,633 ± 3726.424	0.33	1,082,853 ± 4128.5	0.38
4-CQA	1,066,820 ± 6423.833	0.60	1,525,283 ± 6265.7	0.41
3-CQA	1,137,426 ± 6394.687	0.56	80,8061.5 ± 6960.9	0.86
1,3-diCQA	1,454,276 ± 7509.603	0.52	1,106,342 ± 4587.3	0.41
3,5-diCQA	1,743,660 ± 8783.64	0.50	1,958,000 ± 9040.1	0.46
3,4-diCQA	1,723,104 ± 8654.263	0.50	2,705,500 ± 11,548.1	0.43
4,5-diCQA	1,783,382 ± 8864.299	0.49	1,327,115 ± 18,610.1	1.4

**Table 3:** Characterization of degradation products of CQAs determined by HPLC-CID-ESI-MS/MS.

No.	t_R_ (min)	[M − H]^−^	ESI-MS^2^	ESI-MS^3^	Identification	Relative Content *
5-CQA	7.3	353	191	173, 127, 111, 85	5-CQA	74.13%
1 ^b^	10.2	353	191	173, 127, 111, 85	*cis*-5-CQA	24%
2 ^b^	14.4	367	191	173, 127, 85	Methylated 5-CQA	1%
3-CQA	4.6	353	191	173, 127, 85	3-CQA	91.18%
3 ^b^	4.3	353	191	173, 127, 85	*cis*-3-CQA	3%
4 ^a^	7.9	353	173	155, 93	4-CQA	3%
5 ^b^	14.4	335	161	133	Dehydrated 3-CQA	2.8%
4-CQA	7.9	353	173	155, 111, 93	4-CQA	53.91%
6 ^b^	6.6	353	173	155, 111, 93	*cis*-4-CQA	26%
7 ^b^	10.1	367	161	133	Methylated 4-CQA	5%
8 ^b^	13.6	367	161	133	Methylated 4-CQA	10%
9 ^b^	14.3	335	161	159	Dehydrated 4-CQA	5%
1,3-diCQA	11.9	515	353	191, 179, 135	1,3-diCQA	86.97%
10 ^b^	13.3	515	353	191, 179, 135	A *cis*-1,3-diCQA	1%
11 ^b^	13.6	515	353	191, 179, 135	A *cis*-1,3-diCQA	3%
12 ^b^	15.3	515	353	191, 179, 135	Di-*cis*-1,3-diCQA	4%
13 ^a^	16.6	515	353	173, 135	4,5-diCQA	4%
3,4-diCQA	15.4	515	353	173, 135	3,4-diCQA	67.75%
14 ^b^	15.2	515	353	173, 135	*cis*-3,4-diCQA	15%
15 ^b^	16.1	515	353	173, 135	*cis*-3,4-diCQA	12%
16 ^b^	16.5	515	353	173, 135	di-*cis*-3,4-diCQA	6%
17 ^b^	17.6	529	367	161, 193, 135	Methylated 3,4-diCQA	<1%
18 ^b^	18.4	529	367	161, 193, 135	Methylated 3,4-diCQA	<1%
19 ^b^	23.4	497	335	161, 179, 135	Dehydrated 3,4-diCQA	<1%
20 ^b^	24.6	497	335	161, 179, 135	Dehydrated 3,4-diCQA	<1%
21 ^b^	25.8	497	335	161, 179, 135	Dehydrated 3,4-di CQA	<1%
3,5-diCQA	16.4	515	353	191, 135	3,5-diCQA	55.04%
22 ^b^	16.1	515	353	191, 135	A *cis*-1,3-diCQA	43%
23 ^b^	19.1	515	353	191, 135	A *cis*-1,3-diCQA	1%
24 ^a^	7.4	353	191	173	5-CQA	<1%
25 ^a^	15.4	515	353	173, 135	3,4-diCQA	<1%
26 ^b^	21.0	529	367	335, 161, 135	Methylated 3,5-diCQA	<1%
27 ^b^	27.3	543	381	161, 179, 135	Ethylated 3,5-diCQA	<1%
4,5-diCQA	16.6	515	353	173, 135	4,5-diCQA	80.01%
28 ^b^	17.8	515	353	173, 135	A *cis*-4,5-diCQA	<1%
29 ^b^	18.2	515	353	173, 135	A *cis*-4,5-diCQA	5%
30 ^b^	19.0	515	353	173, 135	di-*cis*-4,5-diCQA	12%

^a^: Identified by comparing with reference standards; ^b^: deducted according to reference [16,17,18,19]; * obtained by HPLC-PDA.

## Abbreviations

The following abbreviations are used in this manuscript:

CGAs	Chlorogenic Acids
CQAs	Caffeoylquinic Acids
*p*-CoQAs	*p*-Coumaroylquinic Acids
FQAs	Feruloyl Quinic Acids
HPLC-PDA	High-Performance Liquid Chromatography with Photo Diode Array Detection
HPLC-MS/MS	High-Performance Liquid Chromatography Tandem Mass Spectroscopy
ESI-MS/MS	Electrospray Ionization Tandem Mass
S/N	Signal-to-Noise
